# Emerging roles for fatty acid oxidation in cancer

**DOI:** 10.1016/j.gendis.2024.101491

**Published:** 2024-12-20

**Authors:** Jialin Ma, Shuxian Wang, Pingfeng Zhang, Sihao Zheng, Xiangpan Li, Juanjuan Li, Huadong Pei

**Affiliations:** a Cancer Center, Renmin Hospital of Wuhan University, Wuhan, Hubei 430060, China; b Department of Oncology, Georgetown Lombardi Comprehensive Cancer Center, Georgetown University Medical Center, Washington, DC 20057, USA; c Department of Breast and Thyroid Surgery, Renmin Hospital of Wuhan University, Wuhan, Hubei 430060, China

**Keywords:** Cancer progression, Drug resistance, Fatty acid oxidation, Metabolism reprogramming, Oncotherapy

## Abstract

Fatty acid oxidation (FAO) denotes the mitochondrial aerobic process responsible for breaking down fatty acids (FAs) into acetyl-CoA units. This process holds a central position in the cancer metabolic landscape, with certain tumor cells relying primarily on FAO for energy production. Over the past decade, mounting evidence has underscored the critical role of FAO in various cellular processes such as cell growth, epigenetic modifications, tissue-immune homeostasis, cell signal transduction, and more. FAO is tightly regulated by multiple evolutionarily conserved mechanisms, and any dysregulation can predispose to cancer development. In this view, we summarize recent findings to provide an updated understanding of the multifaceted roles of FAO in tumor development, metastasis, and the response to cancer therapy. Additionally, we explore the regulatory mechanisms of FAO, laying the groundwork for potential therapeutic interventions targeting FAO in cancers within the metabolic landscape.

## Introduction

Metabolic reprogramming serves as a pivotal strategy for meeting the heightened energy and material demands of rapidly proliferating cancer cells.^1^ Among these, the most established metabolic paradigms are aerobic glycolysis and glutamine metabolism.^2^^,^^3^ Additionally, there's a notable increase in fatty acid *de novo* synthesis across various tumors, facilitating the provision of crucial membrane components like phospholipids to sustain the rapid proliferation of tumor cells. However, recent studies have also shed light on the significant upregulation of fatty acid β-oxidation (FAO), the reverse process of fatty acid synthesis (FAS), which plays crucial roles in tumor progression, metastasis, and even drug resistance in a variety of cancer. This apparent paradox can be attributed to the diverse needs of tumor growth; FAS primarily supplies material for cellular components, while FAO serves to provide energy and reducing power.

In this review, we summarize recent advances and insights into the roles of FAO in tumorigenesis and development, as well as its involvement in epigenetics, ferroptosis, tumor microenvironment (TME), and immunotherapy. By elucidating the mechanisms underlying FAO, we lay the groundwork for the development of potential strategies and targets for therapeutic intervention against tumors.

### Biochemical aspects of FAO

**Figure 1:**
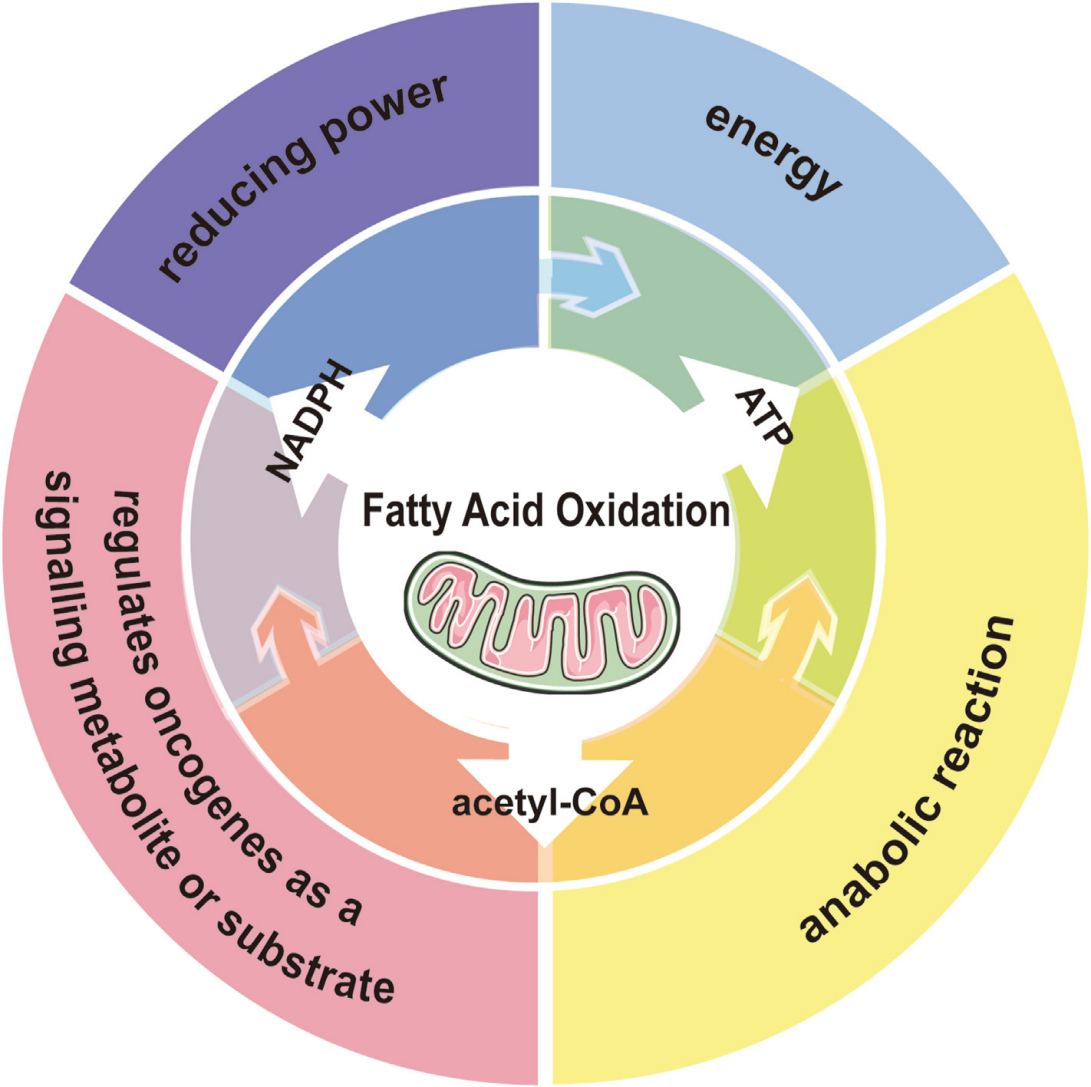
The role of FAO in cancer. FAO provides three essential substances for tumor metabolism, namely acetyl-CoA, NADPH, and ATP. Within the mitochondria, FA undergoes β-oxidation to generate a substantial amount of acetyl-CoA. Acetyl-CoA serves not only as an essential biosynthetic precursor but also as a substrate for acetylation regulation. Acetyl-CoA enters the TCA cycle to generate citrate, which can be transported from the mitochondria to the cytoplasm for its involvement in NADPH production. NADPH serves two pivotal roles; firstly, it counteracts oxidative stress through REDOX reactions, and secondly, it acts as an indispensable anabolic coenzyme facilitating cellular growth and proliferation. FA not only produces ATP during the β-oxidation process, but also contributes to the TCA cycle by metabolizing into acetyl-CoA. The produced ATP not only provides energy but also supports biosynthesis.

**Figure 2:**
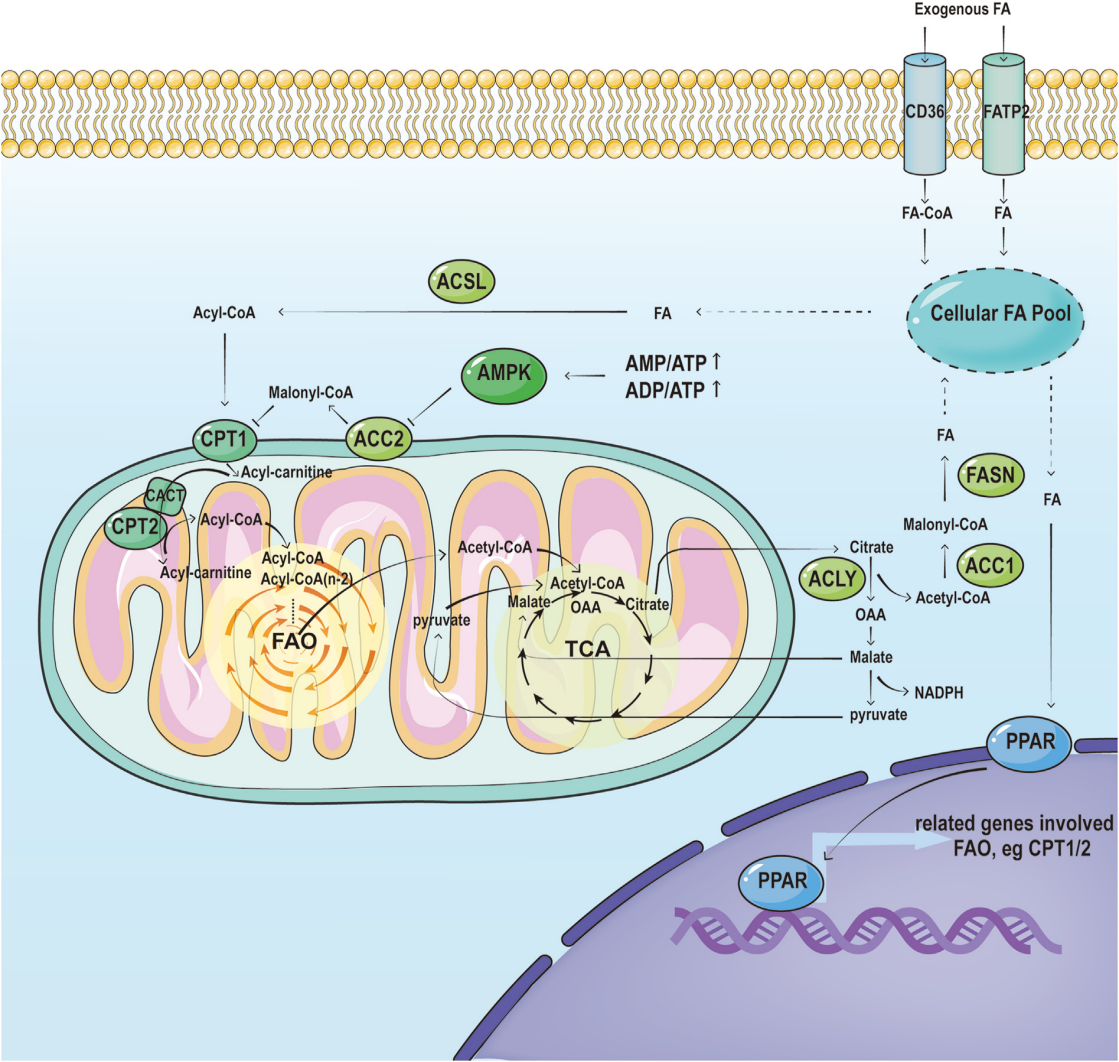
**Fig.2 The process and regulation of FAO**. After being transported into the cell via fatty acid protein transporters, fatty acids are enzymatically converted to acyl-CoA by ACSLs. Subsequently, the carnitine palmitoyltransferase system facilitates the translocation of acyl-CoA from the cytoplasm into the mitochondrial matrix for β-oxidation. The product acetyl-CoA can either enter TCA or exit mitochondria through the citrate-pyruvate cycle. AMPK can regulate ACC activity to alter FAO, and PPAR can regulate the enzyme of FAO at the transcriptional level.

In general, the catabolism of saturated fatty acids (FAs) involves integral three processes: acyl activation, transportation, and β-oxidation. Either exogenous uptake or endogenous *de novo* synthesis, long-chain saturated FAs initially are activated to form acyl-CoA by Long-Chain Acyl-CoA Synthetase (ACSL) in the cytoplasm, with ATP consumption.^4^ Given uncapable of directly pass through mitochondrial membrane, activated acyl-CoA transmembrane shuttle involved in a series of steps as follows: Firstly, carnitine palmitoyl transferase 1 (CPT1), located in the mitochondrial outer membrane, converts acyl-CoA to acyl-carnitine.^5^ Subsequently, acyl-carnitine is transported to the mitochondrial matrix across the membrane by the carnitine/acylcarnitine carrier protein (CACT) in the mitochondrial inner membrane, where carnitine palmitoyl transferase 2 (CPT2) reform acyl-carnitine back to acyl-CoA.^6^^,^^7^ Acyl-CoA access to mitochondria is then gradually degraded step by step referred as to β-oxidation, to produce an acetyl-CoA molecule and two carbons shorter acyl-CoA. Each round of degradation consists of four steps: dehydrogenation, hydration, dehydrogenation, and thiolysis. Acyl-CoA dehydrogenase catalyzes the first step, while the subsequent three steps are catalyzed by the trifunctional protein (TFP) complex, composed of enoyl-CoA hydratase, hydroxyacyl-CoA dehydrogenase, and 3-ketoacyl-CoA thiolase (3-KAT).^8^^,^^9^ It's worth noting that acetyl-CoA acetyltransferase (ACAT1) is responsible for breaking down acetoacetyl-CoA into two molecules of acetyl-CoA during the thiolysis step of the final cycle.^10^ The final product of FAO, acetyl-CoA, either enters the tricarboxylic acid (TCA) cycle followed by oxidative phosphorylation to generate ATP, or is transported into cytosol through the citrate–pyruvate cycle pathway (Fig. 1). Within the cytoplasm, a portion of acetyl-CoA contributes to NADPH production, serving as a carbon source for the biosynthesis of macromolecules, including amino acids, FAs, nucleotides, and steroids, while another portion of cytoplasmic acetyl-CoA enters the nucleus through an acetyl-carnitine shuttle mechanism and participates in histone acetylation^11^ and the transcriptional regulation of target genes such as STATs.^12^ Notably, acetyl-CoA generated by mitochondrial FAO is of crucial importance in hematopoietic stem cells (HSCs).^13^ FAO-derived NADPH contributes to cholesterol synthesis and extracellular vesicle (EV) biogenesis, elevating self-renewal capacity for HSCs,^13^ while FAO-mediated histone acetylation augments HSCs differentiation potential by epigenetically promoting transcription of progenitor cell-related genes.^14^ Beyond histones, acetyl-CoA generated by FAO also facilitates the acetylation of other proteins. For instance, FAO-induced PARP1 acetylation is essential for proper PARP1 activity and DNA damage repair^15^ (Fig. 2).

The β-oxidations for long chain unsaturated FAs (LC-USFA) closely resemble that of long-chain saturated FAs, with a subtle difference: an intermediate specific to LC-USFA, namely cis/trans-3-enoyl-CoA, occurs without the initial dehydrogenation step. Instead, it is essential to convert this intermediate into trans-2-enoyl-CoA using an isomerase to proceed to the subsequent steps.^16^ In comparison to long-chain FAs, a significant portion of short and medium-chain FAs can enter the mitochondrion directly without the need for a translocator. However, short or medium-chain acyl-CoA molecules derived from the breakdown of very long-chain FAs in peroxisomes require trafficking into the mitochondrion through translocases Carnitine O-acetyltransferase (CRAT) and Peroxisomal carnitine O-octanoyltransferase (CROT).^17^, ^18^, ^19^

### Regulation of FAO

As the central part of the metabolic landscape of cancer, FAO is strictly regulated at multiple levels. Under physiological conditions, FAO can be regulated by starvation and some hormones, such as insulin, thyroxine, ERRα and adiponectin. In the following, we will introduce the regulatory mechanisms of FAO at both transcriptional level and posttranscriptional level.

### Transcriptional regulation

Peroxisome proliferator–activated receptor α (PPARα), PPARβ/δ, and PPARγ are a class of ligand-activated nuclear receptors that form heterodimers with retinoid *X* receptor in response to FAs and play key roles in FAO.^20^ PPARα controls the hepatic expression of numerous genes involved in FAO and affects the adaptation of the liver to starvation by enabling the induction of microsomal ω-oxidation, peroxisomal dicarboxylic acid metabolism, and ketogenesis. Similarly, PPARα, along with PPARβ/δ, regulates the expression of FAO enzymes in skeletal muscle and the heart.^21^ PPARs also interact with their transcriptional coactivator PPARγ coactivator-1α (PGC-1α), which binds estrogen-related receptors (ERRs) to control the expression of FAO related enzymes.^22^^,^^23^

Other transcriptional factors, such as PROX1^10^ and sterol regulatory element-binding proteins (SREBPs), can indirectly regulate FAO.^24^ Additionally, the transcriptional coactivator Yes-associated protein (YAP) has been implicated in activating FAO in an unidentified manner.^25^ GFI1B is transcriptional repressor that inhibits the expression for FAO related genes.^26^

### Posttranscriptional regulation

Malonyl-CoA physiologically inhibits FAO through allosterically associating with CPT1A and CPT1B. Cellular malonyl-CoA are produced by acetyl-CoA carboxylase (ACC) and the degradation by malonyl-CoA decarboxylase (MLYCD), thus, both ACC and MLYCD are implicated in FAO regulation. Moreover, the cellular energy sensor AMP-activated protein kinase (AMPK) phosphorylates ACC to inhibit its activity, thereby decreasing malonyl-CoA levels and stimulating FAO.^27^ SIRT2-mediated CPT1α deacetylation enhances CPT1A enzymatic activity and FAO in mouse.^28^ Long chain acyl-CoA dehydrogenase (LCAD), one of the key enzymes catabolizing the first step of β-oxidation, is activated by SIRT3-mediated deacetylation.^29^ Interestingly, fasting-induced acetylation of mitochondrial proteins primarily stems from acetyl-CoA from FAO.^30^ As a result, SIRTs are important for preserving the activity of the key enzymes of FAO.^31^

### Dysregulation of FAO in cancers

As an important source for NADH, NADPH, FADH2, and ATP production, FAO plays a pivotal role in various stages of tumorigenesis, development, and metastasis for numerous cancers, including breast cancer, prostate cancer, glioblastoma, colon cancer, gastric cancer, multiple myeloma, and nasopharyngeal cancer (Table 1). However, it is worth noting that the association of FAO with liver cancers and renal cancers remains controversial.

**Table 1:** The roles of fatty acid oxidation in various cancer types.

Cancer types	Promotion (↑) or suppression (↓)	Functions	Regulatory mechanisms	References
Liver cancer	↓	Hepatocarcinogenesis related to NAFLD	1. CPT2-STAT3 axis induced cell proliferation; 2. CPT2-Src-mediated JNK activation to resist lipotoxicity	^33^^,^^34^
	↓	HCC cell proliferation	YAP-TEAD4 depressed ACADL expression to suppress HCC	^35^
	↑	HCC cell proliferation	HBx upregulates FAO under metabolic stress	^37^
	↑	Chemotherapy resistance to HCC	CPS1 deficiency elevates FAO by activating AMPK	^38^
	↑	HCC progression	Gain-of-function for β-catenin mutant promotes PPARα-mediated FAO	^39^
Breast cancer	↑	TNBC	PKM2-EZH2- SLC16A9-carnitine-FAO	^41^
	↑	TNBC metastasis	CD24-NF-κb-CPT1A signaling pathway	^43^
	↑	TNBC progression	FAO activates YAP signaling and ameliorates oxidation stress	^44^
Ovarian cancer	↑	OC growth and metastasis	Adipocytes provide FAs for FAO and rapid growth in OC cells	^45^
	↓	OC growth and metastasis	CPT2-FAO increases NADPH, which inhibits ROS–NF–κB signaling	^46^
	↑	Platinum resistance	FAO inhibits cell apoptosis	47, 48, 49
Prostate cancer	↑	PCa cell proliferation	Only FAO supply energy for PCa with CDK9 inhibition	^51^
	↑	PCa cell proliferation	Components of carnitine shuttle system are increased	^52^
	↑	The survival of castration-resistant PCa	FAO-acetyl-CoA elevates histone acetylation to activate expression of associated genes	^53^
Renal cancer	↓	CcRCC proliferation	CPT2 activity is reduced	^57^
	↓	CcRCC proliferation	HIF1/2 inhibits CPT1A-FAO and force FAs storage into lipid droplets	^58^
Leukemia	↑	AML cell proliferation	Both very VLCAD and CPT1A are overexpressed	^61^
	↑	Chemotherapy resistance	Combined with FAO inhibitors improves the efficacy	^62^^,^^63^
Lymphoma	↑	Cell proliferation	Mitochondrial trifunctional enzyme subunit alpha (HADHA) is increased	^64^
	↑	DLBCL progression	FAO provides reducing power	^65^^,^^66^
Colorectal cancer	↑	Cell proliferation	circACC1-AMPK-FAO in responds to nutritional and environmental stress	^67^
	↑	Cell proliferation	circACC1-AMPK-FAO in responds to nutritional and environmental stress	^68^
	↑	Metastasis	TGFβ1-SREBP1-ACSL3-FAO mediating EMT while maintaining redox homeostasis	^69^
	↑	Metastasis	FAO inhibits anoikis	^70^
	↑	Metastasis	Enhanced FAO in cancer-associated fibroblasts contribute to metastasis	^71^
GBM	↑	Cell proliferation	Enzymes involved in FAO pathways significant are up-regulate	^72^^,^^73^
	↑	Chemotherapy resistance to GBM	Combined with FAO inhibitors improves the efficacy	74, 75, 76
	↑	Cell proliferation	β-HB/GPR109A-FAO	^77^
	↑	Aggressive growth	FAO promotes CD47-mediated immune escape	^78^
Bladder cancer	↑	Cell proliferation	FAO is significantly increased	^79^
	↑	Metastasis	CPT1B overexpression reduces EMT	^80^
Lung cancer	↑	Cell proliferation	AMPK-FAO	^82^
Nasopharygeal carcinoma	↑	Tumor growth, radio-therapy resistance	CPT1A-FAO-nucleoside metabolism	83, 84, 85

AML, acute myeloid leukemia; AMPK, AMP-activated protein kinase; CcRCC, clear cell renal cell carcinoma; DLBCL, diffuse large B-cell lymphoma; EMT, epithelial–mesenchymal transition; FA, fatty acid; FAO, fatty acid oxidation; GBM, glioblastoma multiform; HCC, hepatocellular carcinoma; NAFLD, non-alcoholic fatty liver disease; OC, Ovarian cancer; PCa, Prostate cancer; TGFβ1, transforming growth factor β1; TNBC, triple-negative breast cancer.

### Liver cancer

Analysis of the TCGA database reveals that the expressions of CPT1 and CPT2 are downregulated in hepatocellular carcinoma (HCC),^32^ suggesting that FAO suppresses the progression of HCC. Remarkably, the downregulation of CPT2 is linked with hepatocarcinogenesis related to NAFLD.^33^ It is reported that steatosis-regulated E2F1 and E2F2 inhibit CPT2 transcription and downstream FAO, which provides a lipid-rich environment for hepatocarcinogenesis.^34^ Mechanistically, on one hand, CPT2 deficiency increases oleoylcarnitine accumulation to motivate STAT3 pathway and cell proliferation, and on the other hand, CPT2 deficiency hinders Src-mediated JNK activation to resist lipotoxicity induced by lipid-rich environment in HCC.^33^ Additionally, ACADL functions as a tumor suppressor in HCC. YAP-TEAD4 directly depress ACADL expression and FAO to promote HCC development.^35^ HIF-1α-mediated suppression of FAO-related enzyme expression impedes apoptosis in HCC, whereas simultaneous inhibition of HIF-1α and activation of FAO can enhance the apoptosis of HCC.^36^

However, others have just the different view. It is shown that hepatitis B virus *X* protein (HBx) facilitates HCC progression by upregulating FAO under metabolic stress.^37^ Similarly, Wu et al discovered that HCC patients with poor prognosis exhibited carbamoyl phosphate synthetase 1 (CPS1) deficiency, which activates AMPK to elevate FAO, consequently rendering chemotherapy resistance to HCC.^38^ Moreover, the gain-of-function for β-catenin mutant responsible for HCC highly relies on PPARα-mediated FAO.^39^

Taken together, the relationship between FAO and HCC progression is still a matter of controversy, which needs much efforts to explore.

### Breast cancer

Mounting evidence is in favor of the notion that FAO is tightly connected with breast cancer progression, drug resistance and metastasis, especially triple-negative breast cancer (TNBC). Glutamine deprivation-elicited HRD1 downregulation maintains CPT2 stability and FAO in TNBC.^40^ Meanwhile, deficiency in glycolysis activates a metabolic switch from glycolysis to FAO to fuel TNBC growth. Inhibition of PKM2 in TNBC leads to impaired recruitment of EZH2 to SLC16A9, which in turn de-represses SLC16A9 expression, thereby increasing intracellular carnitine influx and programming TNBC cells to an FAO-dependent phenotype.^41^ Moreover, cell detachment form matrix is also dependent on FAO, suggesting FAO facilitates TNBC cells metastasis.^42^ TNBC requires a large amount of energy to metastasize, as a result, TNBC reprograms its metabolic pathway from glycolysis to higher energy producing FAO in three manners, including inhibiting AMPK-ACC-malonyl-CoA to upregulating CPT1A, activating PPARα or NF-κB mediated FAO pathway.^43^ Note worthily, to avoid excessive ROS and cell death, FAO elicits YAP pathway to counteract oxidative stress.^44^ Meanwhile, FAO in breast cancer cells commonly is undergone to modulating by adjacent adipocytes and immune cells, see section 5.5 for more details.

### Ovarian cancer

Although *in vitro* and *in vivo* researches have demonstrated that FAs derived from adipocyte breakdown and taken up by cancer cells promote tumor growth in ovarian cancer (OC) by providing energy through β-oxidation,^45^ the role of CPT enzymes in OC remains elusive. However, paradoxically, the expression of CPT2 in OC cells and tumor tissues is significantly lower than non-malignant ovarian cells and paired peritumoral normal tissues, respectively,^46^ even more, low expression of CPT2 is associated with a worse prognosis, perhaps because of G1/G0 cell cycle arrest and increased apoptosis caused by CPT2 overexpression, inhibiting tumor growth and reduced metastasis *in vivo* experiments.^46^ Whereas Perhexiline, a CPT1A/CPT2 inhibitor, has been shown to alleviate platinum-based chemotherapy resistance of OC induced by NKX2-8 loss,^47^ which implies the congruence of CPT1A and CPT2 in chemoresistance, and inhibition of the rate-limiting step of FAO by drugs has also been found to counteract platinum resistance in other studies.^48^^,^^49^ Future studies with larger sample sizes, specific subtypes, and different stages of OC are necessary to explore the impact of FAO net expression on OC.

### Prostate cancer

FAO, rather than glycolysis, constitutes the main bioenergetic process in fatty acid-rich prostate cancer (PCa).^50^ Furthermore, inhibition of CDK9 leads to complete reliance on FAO for energy production in PCa, as evidenced by the synthetic lethal effect of CPT1A when combined with CDK9.^51^ In consistence, multi-omics studies have shown that components of carnitine shuttle system are increased within PCa tissues and cells.^52^ Moreover, the survival for advanced PCa such as castration-resistant prostate cancer (CRPC) is greatly relied on FAO. The acetyl-CoA produced by excess FAO gives rise to elevated histone acetylation which activates expression of genes associated with growth and anti-androgen resistance.^53^ While aiming to counteract with considerable FAO-induced ROS, FAO-caused ER stress sustains a redox balance through serine biosynthesis-folate cycle-and glutathione production axis.^54^

### Renal cancer

Clear cell renal cell carcinoma (ccRCC) is the most common subtype of renal cell carcinoma histologically characterized by abundant lipid deposition in the cytoplasm.^55^ CPT1/2 expression and activity are reduced in renal cancer compared to normal kidney tissue, leading to a poor prognosis for patients.^56^^,^^57^ It has been demonstrated that CPT1A is suppressed by HIF1 and HIF2 in renal cells, resulting in decreased FA transportation to mitochondria and increased FA storage into lipid droplets, which is required for tumorigenesis.^58^

### Leukemia and lymphoma

Acute myeloid leukemia (AML) cells and leukemia stem cells (LSCs) have a specific metabolic phenotype characterized by greater dependence on FAO and oxidative phosphorylation.^59^^,^^60^ It is reported that the very long chain acyl-CoA dehydrogenase (VLCAD) implicated in FAO, is overexpressed and promotes cell proliferation in AML.^61^ Inversely, targeted inhibition of CPT1A enhances the antileukemic activity of ABT199,^62^ and sensitize leukemia cells to cytarabine induced apoptosis.^63^ Of note, recent study has suggested that GFI1B, a transcriptional repressor, hints an excellent prognosis for AML patients on account of epigenetically inhibiting several FAO-related genes.^26^

With regard to lymphoma, FAO-related enzymes are commonly high expressed,^64^ It is shown that mitochondrial trifunctional enzyme subunit alpha (HADHA) is increased in malignant lymphoma tissue,^64^ while HADHB is also an independent predictor of poor prognosis of diffuse large B cell lymphoma (DLBCL).^65^ OxPhos-DLBCL showed enhanced mitochondrial energy metabolism, and FAO interference could effectively inhibit OxPhos-DLBCL.^66^ In DLBCL, the upregulation of FAO coincides with an increase in glycolysis and FA synthesis, which may be due to in fact that some lymphomas grow much faster than other solid tumors and FAO also provides reducing power.^66^

### Colorectal carcinoma

Colorectal cancer cells effectively convert metabolism to FAO to survive in response to metabolic stress or metastasis by sacrificing anabolism. Firstly, inflammation at intestinal tract increases PGE2 levels, promoting FAO and the survival of colorectal cancer cells. Secondly, the increased levels of circACC1, a non-coding RNA spliced from ACC1 RNA, activates AMPK and downstream FAO in colorectal cancer cells, upon nutritional and environmental stress.^67^ FAO also plays a crucial role in driving colorectal cancer, as valosin-containing protein (VCP) enhances the transcription of FAO genes, including CPT1A, by binding to and facilitating the degradation of histone deacetylase 1 (HDAC1).^68^ As for metastasis, transforming growth factor beta 1 (TGFβ1) upregulates ACSL3 responsible for the first step of FAO through the SREBP1 signaling pathway and levels of FAO, mediating epithelial mesenchymal transformation (EMT) as well as metastasis of CRC cells while maintaining redox homeostasis.^69^ The probable mechanism underlying colorectal cancer cell metastasis is to inhibit anoikis by FAO.^70^ In addition, enhanced FAs catabolism in cancer-associated fibroblasts may also contribute to the peritoneal metastasis of colon cancer.^71^

### Glioblastoma multiforme (GBM)

Compared to normal brain tissues, GBM tumors exhibit significant up-regulation of enzymes involved in glucose metabolism and FAO pathways,^72^ consequently, simultaneously inhibiting both pathways can effectively suppress GBM tumor growth,^73^ and either CPT1 inhibitors treatment alone or in combination with other chemotherapy drugs can enhance the anti-tumor effect.^74^^,^^75^ Aurora kinase A inhibitors have emerged as promising drug targets for treating GBM by inhibiting glycolysis through MYC target and PPARα inhibition.^76^ Under favorable nutritional conditions, FAO drives cell proliferation in a β-HB/GPR109A-dependent autocrine manner, while serving as an alternative source of ATP only under unfavorable nutritional conditions.^77^ Enhanced FAs metabolism promotes aggressive growth of GBM accompanied by CD47-mediated immune escape.^78^

### Others

The function of FAO has also been reported in some other tumors. It has been reported that the level of free fatty acid (FFA) and FAO were significantly higher in bladder cancer tissues than in adjacent tissues, in concert, etomoxir could inhibit the growth of bladder cancer cells both *in vitro* and *in vivo*.^79^ However, confusingly, some studies have found that the down-regulation of FAO-related protein CPT1B contributes to high-grade bladder cancer, vice versa, overexpression of CPT1B in high-grade bladder cancer cells can reduce EMT *in vitro*, and diminish cell proliferation, EMT and metastasis *in vivo*.^80^ ATP production in pancreatic ductal adenocarcinoma is dependent on fatty acid oxidation rather than glycolysis.^81^ The activation of AMPK and the enhancement of FAO were observed in lung cancer cells under glucose restriction.^82^ As for nasopharyngeal carcinoma (NPC), FAO is implicated in both tumor growth and radiotherapy resistance.^83^, ^84^, ^85^

### The mechanisms by which FAO regulates cancer

#### FAO, metabolic reprogramming and cancer cell growth

Energy supply and building blocks are primarily dependent on glucose and glutamine rather than FAs in solid tumors, even though FAO proves more efficient than glucose-derived pyruvate oxidation at generating ATP per nutrient molecule. Indeed, glutamine is sufficient for sustaining cell growth in glucose-deficient colorectal cancer cells while FAs fail to do so.^86^ It seems that FFAs exhibit minor abilities compared to glucose, even glutamine, when it comes to supply tumors with bioenergy for cell proliferation. However, FAO can supply ATP at a required rate for supporting cancer proliferation but solely as an adaptive response towards the extracellular acidic environment.^87^ To a great extent, FAO contributes to counteracting the acidic virulent environment produced by tumors. Excessive glycolysis-induced lactate accumulation can acidify both the cytoplasm and extracellular space, inhibiting tumor growth via ROS-triggered-cell death and negative feedback inhibition of glycolysis. Hence, switching glucose metabolism to FAO is potent to relieve cellular acidosis.

However, there are exceptions. A subset of B-cell lymphomas utilizes mitochondrial FAO as their primary metabolic strategy for anabolism and proliferation. In these tumors, the upregulation of FAO coincides with FAs synthesis,^66^ which is necessary to meet the unique high ATP demand due to a subgroup of lymphoma that grows much faster than other solid tumors. Additionally, FAO provides mitochondrial reducing power required for the antioxidant glutathione to effectively combat increased ROS production caused by B-cell lymphomas suspension growth.

In addition to providing ATP to support cancer proliferation, FAO-related enzymes or products may also aid proliferation by inhibiting signals that block proliferation. PKM2 is a specific subtype expressed by cancer cells. PKM2 increases glycolytic flux and reduces the dependence on mitochondrial oxidative phosphorylation for proliferation.^88^ PKM2 activity maintains its sensitivity to glucose levels and is activated by the glycolytic intermediate fructose 1, 6-diphosphate (F-1, 6-BP). Although F-1, 6-BP activates PKM2, high concentrations of F-1, 6-BP can reduce CRC proliferation,^89^ in which case a decrease in PFK1 activity caused by FAO-derived citrate will prevent F-1, 6-BP from increasing to levels that prevent proliferation.

### FAO and epigenetics

The epigenetic change is another hallmark of cancer. Compared to classical genetic regulation, epigenetic modulation is more flexible and variable, rapidly responsing to numerous stimuli, thus allowing tumor cells to adapt to their surrounding alterations.^90^ Acetyl-CoA derived from FAO serves as a substrate for acetylation, which is involved not only in the acetylation of proteins,^12^^,^^91^ but also in the acetylation of histones. For example, Acetyl-CoA derived from FAO enhances H3 and H4 acetylation, regulating anti-tumor polarization in macrophages.^92^ Conversely, epigenetic changes are involved in cancer advancement by regulating FAO. Glucose metabolism deficiency blocks histone methyltransferase EZH2 recruitment, attenuating epigenetic silencing of the carnitine efflux transporter SLC16A9 and increasing FAO and TNBC growth.^41^

### FAO and ferroptosis

Ferroptosis is a form of programmed cell death caused by ROS-induced peroxidation of polyunsaturated fatty acids (PUFA) in a ferric ion dependent manner. Previous studies have identified ferroptosis as a natural tumor suppression mechanism, whose inactivation, similarly to apoptosis inactivation, contributes to tumor development and drug resistance. In addition to mGPX4 and DHODH in mitochondria eliminating ROS, tumor cells commonly exploit various antioxidant pathways including GSH/GPX4 axis, FSP1/CoQ10 system and GCH1/BH4/DHFR system, all of which rely heavily on NADPH. Surprisingly, there is no evidence that tumor cells leverage FAO-produced NADPH to prevent ROS-induced ferroptosis. However, Acetyl-CoA stemmed from FAO fosters TCA cycle and cell respiration under acidosis, and block complex I activity and ROS production by hyperacetylation for mitochondrial non-enzymatic proteins, which suppresses apoptosis and ferroptosis.^93^ Cytotoxic T lymphocyte subset 9 (Tc9) reduces lipid peroxidation and declines ferroptosis stress to durably kill tumor cell through STAT3-mediated FAO upregulation.^94^ Two studies in ccRCC suggest that PUFA peroxidation and ferroptosis are fostered by FAO-induced ROS.^95^^,^^96^ Nevertheless, the relationship between FAO and ferroptosis in other cancers still requires for further study.

### FAO, protein palmitoylation and cell signaling transduction

**Figure 3:**
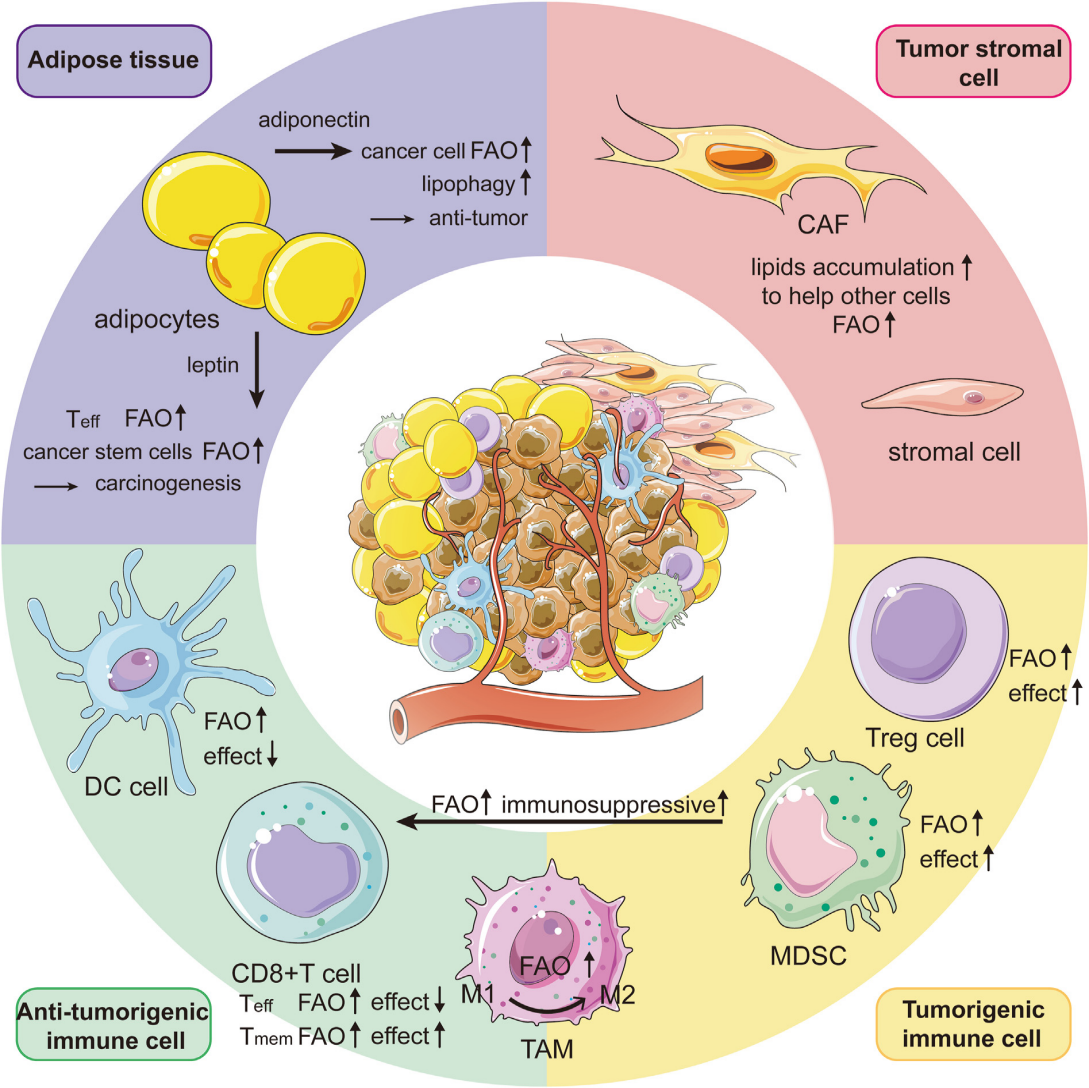
FAO in the TME. A variety of cells in TME alter their own or other cell functions through abnormally activated FAO. For example, adipocytes activated by tumors secrete factors such as leptin and adiponectin to enhance the FAO in tumor or immune cells within the TME. In addition, fatty acids synthesized and accumulated in CAFs and stromal cells activated by tumor cells are utilized by tumor cells to enhance FAO and promote tumor progression. Moreover, FAO transforms immune cell phenotypes and functions; most typically, TAM cells tend to favor the tumor-promoting M2 phenotype as FAO increases. An increase in FAO in DC cells and Teff cells could promote their survival but does not exert an anti-tumor effect. However, FAO could help Tmem cells survive. In summary, FAO was activated and altered its function in a variety of cells in the TME.

Protein palmitoylation is a widespread lipidation modification with attaching palmitoyl group to cysteine residues on substrate protein via thioester bonds.^97^ Protein palmitoylation regulates a large variety of oncogene-coded protein trafficking, stability and functions, which are implicated in cell signaling transduction and tumorigenesis. Coincidentally, Palmitoyl-CoA synthesized by ACSLs is not only the upstream raw material for FAO, but also serves as the donor for protein palmitoylation (Fig. 3), therefore in cancer, enhanced FAO passively increases palmitoylation levels for protein involved in signaling transduction pathways including MAPK, hippo to promote cancer progression.^98^^,^^99^ Consistently, FASN mediated palmitic acid *de novo* synthesis not only regulates FAO, but also affects protein palmitoylation, however, the crosstalk between the FASN-ACSL-FAO axis and FASN-ACSL-palmitoylation axis and their contributions to cancer remains to be further explored.

### FAO and tumor microenvironment

**Figure 4:**
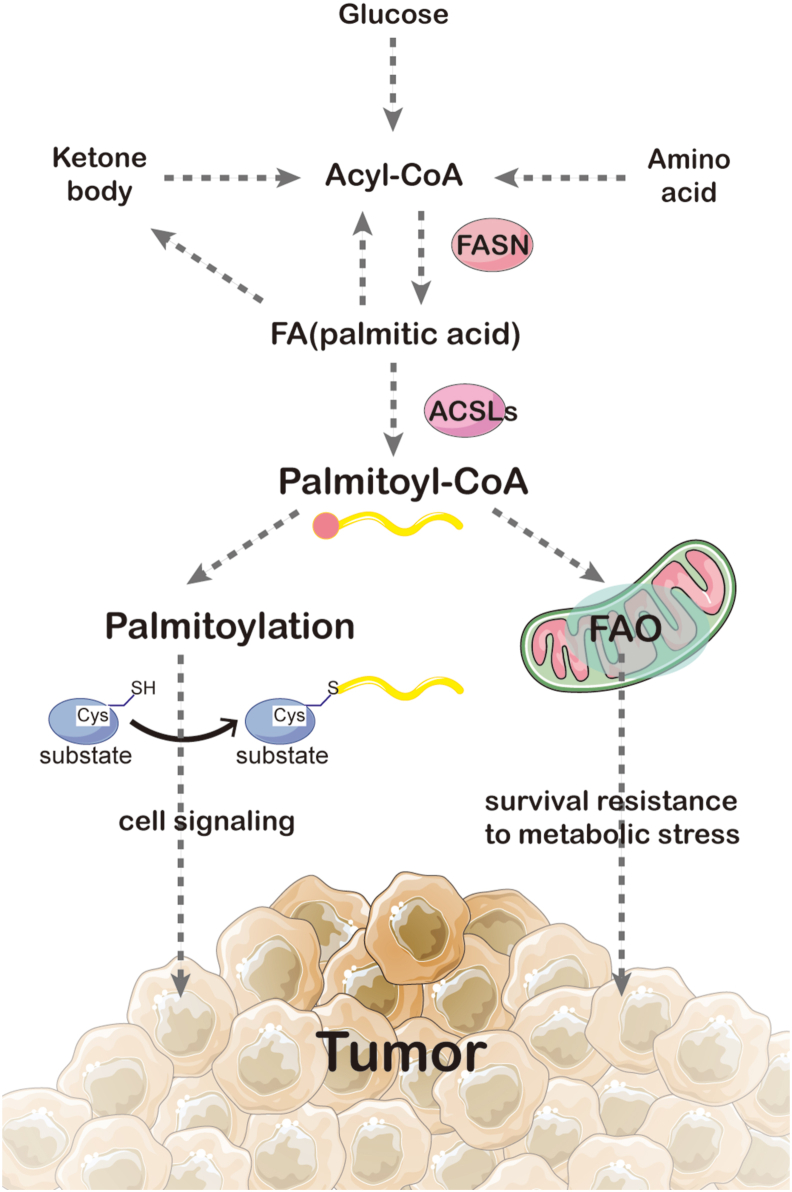
Crosstalk between FAO and protein palmitoylation. Besides being involved in plasma membrane assembly in rapidly proliferating cells, FASN-synthesized fatty acids are activated by ACSLs to their active form, acyl-CoA. Acyl-CoA is partially transported to mitochondria, where it participates in FAO and acetyl-CoA production to meet energy demands. Meanwhile, the remainder is involved in protein acylation, specifically palmitoylation, which promotes cancer progression through signal transduction, including the Wnt, MAPK, TGF-β, and Src pathways.

It is well known that various cells surrounding the tumor, including immune cells, adipocytes, cancer-associated fibroblasts (CAFs), stroma, and extracellular matrix, create conducive and protective niches for tumor survival, proliferation, and metastasis, referred to as TME. The formation of TME is closely reliant on reprogrammed metabolic pathways in tumor-associated immune cells. For example, leptin produced by adipocytes upregulates enzymes involved in FAO by activating the transcription factor STAT3, which inhibits the glycolysis of CD8^+^T effector cells and impair their anti-tumor effects.^100^ Conversely, memory T cells (T_mem_) show a tendency to exploit FAO to enhance their function.^101^ It is indicated that tumor necrosis factor receptor-associated factor 6 (TRAF6) in CD8^+^ *T*_mem_ cells shifts metabolic pathway towards FAO, thereby enhancing the generation of memory cells and protective immunity.^102^ (Fig. 4).

FAO is altered not only in CD8^+^ T cells, but also in CD4^+^ T cells. PD-1 ligation signal switches metabolism from glycolysis and amino acid catabolism to FAO through blocking the PI3K/Akt and MEK/ERK pathways and upregulating CPT1A and ATGL to sustain survival in CD4^+^ T cells.^103^ Whether regulatory T cells (T_reg_s) depend on FAO remains controversial. On one hand, Th1, Th2, and Th17 cells rely more on glycolysis and *de novo* fatty acid synthesis to support effector functions, whereas T_reg_s rely more on oxidative phosphorylation and FAO.^103^^,^^104^ One study showed that in glioblastoma, HIF-1α directs glucose away from mitochondria and enables T_reg_ s mitochondrial FAO, enhancing the immunosuppressive capacity of T_reg_s.^105^ The activation of Several aryl hydrocarbon receptor (AhR) promotes the generation of T_reg_s by enhancing Lkb1-mediated FAO through the Skp2/K63-ubiquitination pathway.^106^ During iT_reg_ differentiation stimulated by transforming growth factor β1 (TGFβ1), the ubiquitination and subsequent degradation of ATP-citrate lyase (ACLY) facilitate the differentiation process of FAO and iT_reg_^107^. On the other hand, Treg cell differentiation was not dependent on CPT1A expression, suggesting that FAO is not indispensable for T_reg_ cell function.^108^ Further investigations are warranted to elucidate the specific lipid molecules or metabolic processes involved in FAO that play a critical role in T_reg_ cell differentiation.

With regarding to myeloid cells in TME, FAO enhances the ability to suppress T cell responses for myeloid-derived suppressor cell (MDSC), while it impairs anti-tumor effects for dendritic cells (DCs), as observed by significantly increased FAO-related catabolic pathways in tolerant DCs.^109^ Unlike glycolysis in M1 macrophages with anti-tumor function,^110^ FAO is dominant in M2 macrophages. This is exemplified by tumor-associated macrophages (TAM) for hepatocellular carcinoma (HCC), in which RIPK3 is down-regulated and attenuates ROS and caspase1-mediated PPAR cleavage, which enhanced PPAR-dependent FAO.^111^ FAO facilitates IL-1β release in TAM, contributing to HCC migration.^112^

Besides supplying FFAs through lipolysis,^113^, ^114^, ^115^, ^116^, ^117^ adipose tissue surrounding tumors also releases other factors that stimulate cancer progression. Leptin secreted from adipocytes activates JAK-STAT3 signaling pathway in breast cancer stem cells (BCSCs), which heightens FAO via STAT3 association with CPT2 promoter, contributing to tumor spheroid forming.^118^ In comparison, adiponectin from adipocytes takes on an inhibitory role in the cell growth for breast cancer via stimulation of lipophagy-mediated lipolysis and FAO.^119^^,^^120^

### FAO and metastasis

During metastasis, cancer cells rely on FAO under nutrient and oxygen-deficient environmental conditions. Tumor cells undergo EMT in the TME, migrate through lymph nodes and blood to achieve distant metastasis, developing a stronger invasion ability and establishing a survival space. Currently, the majority of solid tumor patients succumb to tumor metastasis, with numerous studies highlighting the pivotal role of FAO in this process.

### EMT

EMT is involved in stemness, invasion, migration, anti-apoptosis, and metabolic reprogramming in tumors. It is a reversible cellular process which facilitates the local invasion of cancer cells into adjacent tissues and subsequently enables their infiltration into blood vessels or lymphatics, and is a prerequisite for metastasis. Increasing evidence has shown that FAO enhances EMT in various tumors. Firstly, FAO stimulates EMT by acetylating and activating the FABP12-PPARγ pathway, leading to PCa metastasis.^121^ Secondly, FAO promotes mitochondrial fission and EMT gene expression, thereby activating HCC cell migration.^122^ Consistently, FAO elevates mitochondrial ROS levels, activating the p38 mitogen-activated protein kinase (MAPK) signaling pathway and inducing EMT in tumor ball cells with high ROS expression, ultimately enhancing tumor invasion and metastasis *in vitro*.^123^ In gastric cancer tissues, overexpression of CPT1A promotes metastasis by upregulating the expression of EMT marker proteins Vimentin and Snail while reducing the expression of E-cadherin^124^. Interestingly, EMT also in turn enhances FAO activity alongside oxidative phosphorylation through the p-AMPK pathway.^125^ Co-culturing colon cancer cells with adipocytes induces EMT via downregulation of E-cadherin expression coupled with upregulation of Vimentin expression.^126^ Additionally, ACSL3-mediated FA oxidation is essential for TGFβ1-induced EMT and colorectal cancer metastasis.^69^ Retinoids can reverse EMT to mesenchymal-epithelial transformation (MET) by redirecting the utilization of FAs from β-oxidation in the stromal cell state towards lipid storage in the epithelial cell state. This occurs through binding to genes that specifically target lipid anabolism.^127^

### Lymph node metastasis

Lymph nodes are key places of tumor-immune cell interaction and potential channels for tumor cell metastasis to other sites, which enter the systemic circulation via thoracic ducts or lymphatic vessels.^128^ A study in *Science* Journal has shown that metastatic tumor cells probably prefer to utilize FAs as an energy source in lipid-rich lymph nodes.^25^ By comparing the transcriptomics and metabolomics of primary and LN metastatic tumors in mice, this study found cell metabolism shift to FAO is required for LN metastasis,^47^ in which the accumulated bile acids in the metastatic lymph nodes may selectively activate the transcriptional coactivator YAP in tumor cells through vitamin D nuclear receptors, resulting in the upregulation of gene expression in the FAO signaling pathway. In consistency, both pharmacological inhibition of FAO and gene inhibition of YAP could inhibit lymph node metastasis in mice.

Primary tumors are capable of stimulating lymphangiogenesis, leading to metastasis and inflammation.^129^ A study in *Nature* Journal showed that FAO is a metabolic regulator of lymphangiogenesis.^11^ FAO promotes nucleotide synthesis to support DNA replication and mediates epigenetic changes in histone acetylation, contributing to the transcription of key lymphoid genes, promoting veno-lymphatic endothelial cell differentiation, and thereby facilitating lymphatic metastasis. In addition, Wong et al revealed that genetic and biochemical disruption of CPT1A function inhibits lymphangiogenesis,^11^ and LN metastasis requires a metabolic shift towards FAO.^130^ Acetyl-CoA produced by FAO can acetylate histones, and histone acetylation is particularly important for epigenetic regulation of gene expression related to lymphangiogenesis.^131^ The metastasis of LN necessitates a metabolic shift towards FAO that is induced by MITF in Acral melanoma cells.^132^ Ferroptosis is a formidable challenge for circulating tumor cells (CTCs) to metastasize to distant organs. Oleic acid may protect CTCs from the threat of ferroptosis by reducing the amount or density of polyunsaturated fatty acids used for oxidation in the membrane, as observed in melanoma.^133^

### Distant metastases

Accumulating evidence supports the presence of tendentious changes to FAO of metastasizing cells, contributing to their ability to successfully colonize in the distant organs. Anoikis is a form of special apoptosis resulting from detachment from extracellular matrix and a significant role in distant metastasis. CPT1A-mediated FAO promotes metastasis of colorectal and OC cells by inhibiting anoikis.^70^^,^^134^ CPT1A inhibition of CRC cells within the lung engraftment rate is far lower than the normal CRC cells.^70^ Studies have found that liver metastases of pancreatic ductal adenocarcinoma (PDAC) exhibit drug resistant stem cells and EMT-like phenotypes, accompanied by metabolic phenotypes mediated by aerobic glycolysis and fatty acid β-oxidation.^135^ In the TME, enhanced FAs catabolism of cancer-associated fibroblasts can drive peritoneal metastasis of colon cancer.^71^

### FAO and immunotherapy

Targeting FAO in combination with immunotherapy, including immune checkpoint blockade and chimeric antigen receptor-engineered human T cells (CAR-T), has emerged as a promising treatment strategy. For instance, enhanced FAO and TCA in DCs caused by paracrine Wnt-β-catenine-PPARγ-CPT1A axis signaling induces protoporphyrin IX biogenesis and indoleamine 2, 3-dioxgenase-1 (IDO) activation, leading to T_reg_ cells differentiation through mediating tryptophan catabolism, ultimately promoting melanoma immune escape.^136^ Thus, suppressing FAO in DCs predominantly improves the benefit for anti-PD-1 therapy. In addition, IFN-γ released by effector T cells enhances FAO in tumors through eliciting CPT1A expression, activating pro-survival signaling and antagonizing cytotoxic effects. Therefore, blocking FAO also ameliorates the benefit for immunotherapy with CAR-T.^137^ However, different views exist in the field. For instance, in response to glucose and oxygen depletion, CD8^+^ tumor-infiltrating lymphocytes (TILs) maintain energy production and function via PPAR-α-mediated FAO; as a result, increasing FAO in TILs and synergy with anti-PD-1 therapy achieves better efficacy.^138^^,^^139^

### FAO and cancer drug resistance

Drug resistance has become a significant challenge for therapy, leading to tumor relapse and refractory. An increasing number of studies have shown that the aberrant activation of FAO is one of the major causes for tumor drug resistance, observed in breast cancer, nasopharynx cancer, gastric cancer, leukemia, and PCa with drug resistance.^70^^,^^84^^,^^118^^,^^140^^,^^141^ For instance, tumor cells resistant to cisplatin produce more reducing power through augmented β-oxidation, coupled with decreased lipogenesis, to respond to cisplatin-induced oxidative stress.^142^ Li and colleagues have demonstrated that substantial acetyl-CoA generated by FAO results in increased phospholipid synthesis to sustain mitochondrial integrity, protecting tumor cells from chemotherapy-induced apoptosis.^12^ Dexamethasone triggers increased PPARα and enhanced FAO levels to resist glucocorticoid-mediated cytotoxic effects.^143^ Additionally, mesenchymal stem cells (MSC)-derived lncRNA induces chemotherapy resistance by regulating mitochondrial β-oxidation in gastric cancer and breast cancer.^144^^,^^145^ Similarly, reprogramming of arachidonic acid catabolism confers glioblastoma resistance to temozolomide.^146^ Loss of NKX2-8 leads to increased FAO in ovarian epithelial cells with platinum resistance in the fat microenvironment. Remarkably, pharmacological inhibition of the FAO pathway with perhexiline elevates the therapeutic effect of platinum drugs on ovarian cancer.^47^ The NPRA protein safeguards Mfn2 against degradation, facilitating its localization to mitochondria, thereby enhancing FAO and consequently promoting stemness and chemoresistance.

Abnormal FAO upregulation not only confers resistance to chemotherapy but also to radiotherapy. Radiation-resistant breast cancer cells generate increased FAO and ATP production, leading to hyperactivation of MAPK signaling and decreased apoptosis, resulting in a more aggressive phenotype.^147^ In nasopharyngeal carcinoma, Rab 14 association with CPT1A promotes the FA transport from lipid droplets to mitochondria and β-oxidation, reducing radiation-induced lipid accumulation and apoptosis.^84^ In radioresistant GBM tumors, CPT1A and CD47 are upregulated through NF-κb/RelA acetylation by FAO-produced acetyl-CoA.^78^

### Therapeutic interventions for targeting FAO

The differential dependency on FAO between cancerous and normal tissue provides a significant therapeutic opportunity to target cancer cells while minimizing side effects on normal cells. Currently, effective therapeutic strategies for inhibiting FAO primarily focus on targeting its rate-limiting step, specifically the CPTs transport system.

Etomoxir is a glycidyl ester derivative that is metabolized and converted to the corresponding CoA esters in the body.^148^ Its partially oxidized ring covalently binds to and directly acts on the active site of CPT1, irreversibly inhibiting CPT1A and CPT1B.^5^ While some studies have reported increased chemotherapeutic benefits when etomoxir is combined with chemotherapy, a phase II clinical trial for treating type 2 diabetes was discontinued due to liver and heart side effects.^149^ ST1326 is an analogue of palmitoyl carnitine and a physiological substrate of CPT2,^150^^,^^151^ which has strong cytotoxicity and significantly increases apoptosis in lymphoma and leukemia cells by blocking FAO.^62^^,^^152^ Perhexiline and Ranolazine were originally developed as anti-angina drugs that diminish FAO by inhibiting CPTs.^153^, ^154^, ^155^, ^156^ However, the mechanism underlying tumor inhibition is complicated and does not solely rely on FAO.

In addition to targeting CPTs, several drugs targeting other enzymes modulating FAO pathway are under development. For instance, Triacsin C inhibits ACSLs activity to hinder the production of palmitoyl-CoA, a substrate for palmitic acid oxidation. This induces cell apoptosis in lung cancer, colon cancer, and brain cancer cells.^157^ 2-bromopalmitate, an analog of palmitic acid, competitively binds with CPT1 to exert inhibitory effects. Of note, these two processes may be associated with tumor procession through involvement in palmitoylation rather than FAO.^158^

Moreover, FAO contributes to tumor resistance to various drugs such as platinum, paclitaxel, dexamethasone, l-asparaginase, cytarabine, and tamoxifen. Therefore, the combination of FAO-inhibiting drugs with first-line treatments often yields better clinical efficacy. For example, utilizing the FAO inhibitor perhexiline may enhance the efficacy of chemotherapy with oxaliplatin for gastrointestinal cancers.^153^ Etomoxir can sensitize tumor cells to chemotherapy drugs such as cisplatin.^159^^,^^160^ In a syngeneic glioma model (oncogenic neural stem cells), etomoxir increased survival,^161^ while its combination with glucose analog and glycolytic inhibitor 2-deoxy-d-glucose (2-DG) led to metabolic lethality *in vitro* and increased median survival in mice bearing MES93 mesenchymal GBM tumors.^77^

Although FAO inhibitors have been used in clinical practice for heart disease,^66^^,^^162^ their application in tumors is still in the preclinical stage, partly due to drug toxicity. For instance, Oxfenicine can also restrain CPT1 activity, but has not been studied in tumors.^163^ On the other hand, FAO can be indirectly activated by PPAR activators, AMPK activators, or ACC inhibitors, so inhibiting these targets may also inhibit FAO, but it may also affect other pathways. Traditional platinum drugs were modified to interact with CPT1A to inhibit FAO, thereby enhancing the anti-tumor effect of platinum drugs.^141^ At the same time, there are many drugs with unknown targets that may modulate FAO, such as high-dose dexamethasone, which can delay tumor growth and promote apoptosis by inhibiting CPT1A,^164^ and metformin, which also inhibits FAO in breast cancer.^165^

## Conclusion

Until recently, the Warburg effect dominated discussions on cancer metabolism, overshadowing other crucial molecular mediators. Initially, the misconception surrounding the Warburg effect arose from the belief that malignant cells acquire their glycolytic conversion ability through mitochondrial defects.^166^^,^^167^ While this concept advanced our understanding of cancer development, more recent evidence underscores the pivotal role of mitochondrial function in cancer, including transformation and drug resistance. Here, we delve into the impact of β-oxidation on tumors, dissecting the specific mechanism driving FAO in various tumor types or molecular subtypes. PCa with limited glycolytic capacity, and breast, ovarian, and colorectal cancers growing in fat-rich environments are more likely to rely on overactivation of FAO. However, FAO is consistently inhibited in liver cancer and ccRCC. Since the liver is vital for fat metabolism processes, adipogenesis induced by FAO inhibition promotes cancer development through proto-oncogene activation, while down-regulating related pathways can inhibit cell death caused by lipid toxicity. In ccRCC, FAO inhibition may result from the stabilization and transfer of HIF protein to the nucleus following hypoxia or deletion of VHL. The metabolic adaptation of ccRCC results from multiple metabolic abnormalities mediated by HIF. On one hand, HIF promotes FAS; on the other hand, it inhibits CPT1A to impede FAO, leading to further accumulation of cytoplasmic lipids and promoting the occurrence and development of ccRCC.^55^^,^^58^ Although there are variations in the impact of FAO on tumor growth, it is undeniable that tumor metastasis tends to exploit abnormally activated FAO, introducing a novel perspective into the study of tumor metastasis and offering potential targets for inhibiting metastasis.

**Figure 5:**
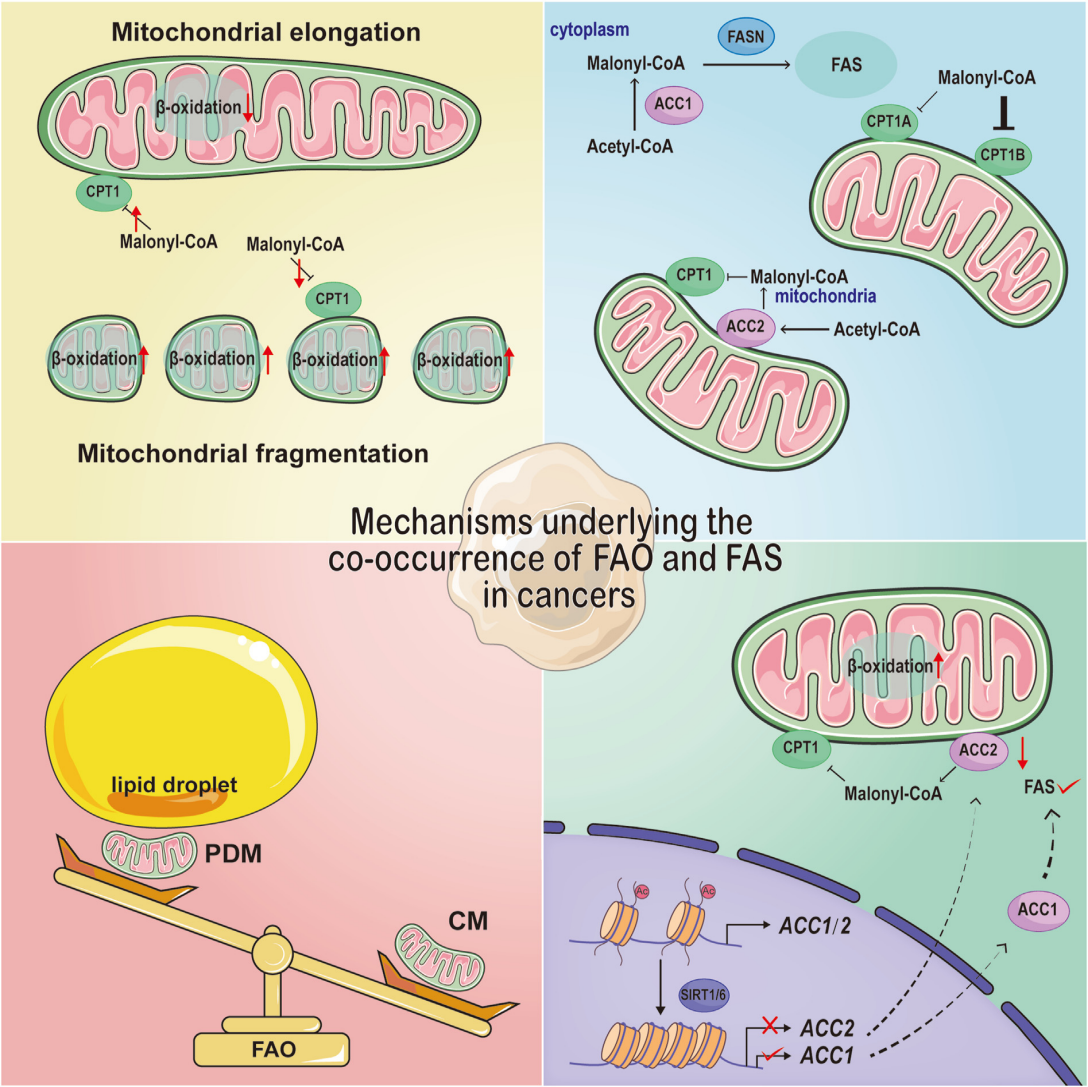
Mechanisms underlying the co-occurrence of FAO and FAS. 1) Mitochondrial fragmentation reduces malonyl-CoA-mediated inhibition of CPT1 and increases FAO; in contrast, mitochondrial elongation attenuates FAO. 2) Different CPT1 isoforms exhibit varying sensitivity to malonyl-CoA, and malonyl-CoA produced by ACC1 in the cytoplasm fails to inhibit FAO. 3) FAO in peridroplet mitochondria (PDM) is weaker than that in cytoplasmic mitochondria (CM). 4) Sirtuin-mediated histone deacetylation selectively decreases mitochondrial Malonyl-CoA-producing ACC2 transcription but not ACC1-mediated FAS. The above mechanisms enable the enhancement of FAO in FAS-enhanced tumor cells.

Tumor cells require proliferation and synthesis of diverse FAs to generate biomembranes structures. Therefore, cancer cells often exhibit upregulated adipose *de novo* formation. Current research also focuses on the effects of FAS on tumors. However, the influence of FAO on tumors has been overlooked due to malonyl CoA's ability to inhibit FAO as a product of FAS. With increasing studies on FAO, we have come to realize that tumor cells can simultaneously engage in both FAO and FAS processes. The phenomenon can be attributed to several mechanisms: firstly, two types of Malonyl-CoA produced in the cytoplasm and on the mitochondrial outer membrane respectively promote FAS while inhibiting FAO. Additionally, different subtypes of CPT1 display varying sensitivities towards inhibition by Malonyl-CoA. Recently discovered evidence suggests that peridroplet mitochondria (PDM) experience decreased β-oxidation possibly due to segregated mitochondrial populations enabling simultaneous occurrence of both FAO and FAS.^168^ Furthermore, mitochondrial fragmentation reduces CPT1 sensitivity towards malonyl-CoA inhibition while enhancing long chain fatty acid oxidation through increased FAO. Conversely, elongation increases the sensitivity of CPT1 towards malonyl-CoA inhibition.^169^ It has also been observed that Sirtuin-mediated histone deacetylation, coupled with continuous down-regulation of ACC2, enables compatibility between mitochondrial fatty acyl-CoA and intracellular lipid production, thereby facilitating the simultaneous occurrence of both FAO and FAS. Corbett et al also discovered that FAO-induced increases in acetyl-CoA result in alterations to histone acetylation within the nucleus, selectively reducing transcription of ACC2 but not ACC1.^93^ This selective reduction of ACC2 provides a mechanism for simultaneous FAO and FA synthesis in stressed cells (Fig. 5).

Currently, our understanding of the relationship between FAO and tumors is limited to enzymes involved in rate-limiting steps such as CPT1/CPT2. However, the comprehensive and systematic regulatory network of FAO and its role in tumorigenesis is still not clear. Additionally, the structure of key FAO enzymes like CPT1 is still speculative at this stage. We believe that determining the structure of this membrane protein would significantly enhance our understanding of FAO regulation, thereby laying the foundation for the design of specific inhibitors for cancer treatment. Furthermore, there is significant heterogeneity in cancer metabolism, particularly regarding FAO which exhibits more flexibility compared to the well-known Warburg effect and increased glutamine consumption. Due to its differential expression between tumor cells and normal cells, targeting FAO can offer a therapeutic window with minimal side effects on normal cells, providing an opportunity for targeted therapy. Combining FAO targeting with other anti-tumor therapies may hold the key to controlling tumor progression and metastasis effectively.

## CRediT authorship contribution statement

**Jialin Ma:** Visualization, Writing – original draft. **Shuxian Wang:** Visualization. **Pingfeng Zhang:** Supervision. **Sihao Zheng:** Conceptualization, Writing – original draft, Writing – review & editing. **Xiangpan Li:** Supervision. **Juanjuan Li:** Supervision. **Huadong Pei:** Conceptualization.

## Conflict of interests

The authors declare no competing interests.
